# The common H232 STING allele shows impaired activities in DNA sensing, susceptibility to viral infection, and in monocyte cell function, while the HAQ variant possesses wild-type properties

**DOI:** 10.1038/s41598-023-46830-5

**Published:** 2023-11-09

**Authors:** Guendalina Froechlich, Arianna Finizio, Alessandra Napolano, Sara Amiranda, Arianna De Chiara, Pasqualina Pagano, Massimo Mallardo, Guido Leoni, Nicola Zambrano, Emanuele Sasso

**Affiliations:** 1 Dipartimento di Medicina Molecolare e Biotecnologie Mediche, Università degli Studi di Napoli Federico II, Via Pansini 5, 80131 Napoli, NA Italy; 2 CEINGE Biotecnologie Avanzate Franco Salvatore S.C.aR.L., Via Gaetano Salvatore 486, 80145 Naples, Italy; 3 Nouscom S.R.L., Rome, Italy; 4 ImGen-T Srl, Viale del Parco Carelli, Napoli, NA Italy

**Keywords:** Infection, Innate immunity

## Abstract

Different innate immune pathways converge to Stimulator of interferon genes (STING) and trigger type I interferon responses after recognition of abnormal nucleic acids in the cells. This non-redundant function renders STING a major player in immunosurveillance, and an emerging target for cancer and infectious diseases therapeutics. Beyond somatic mutations that often occur in cancer, the human gene encoding STING protein, *TMEM173 (STING1)*, holds great genetic heterogeneity; R232, HAQ (R71H-G230A-R293Q) and H232 are the most common alleles. Although some of these alleles are likely to be hypomorphic, their function is still debated, due to the available functional assessments, which have been performed in biased biological systems. Here, by using genetic background-matched models, we report on the functional evaluation of R232, HAQ and H232 variants on STING function, and on how these genotypes affect the susceptibility to clinically relevant viruses, thus supporting a potential contributing cause to differences in inter-individual responses to infections. Our findings also demonstrate a novel toll-like receptor-independent role of STING in modulating monocytic cell function and differentiation into macrophages. We further supported the interplay of *STING1* variants and human biology by demonstrating how monocytes bearing the H232 allele were impaired in M1/M2 differentiation, interferon response and antigen presentation. Finally, we assessed the response to PD-1 inhibitor in a small cohort of melanoma patients stratified according to STING genotype. Given the contribution of the STING protein in sensing DNA viruses, bacterial pathogens and misplaced cancer DNA, these data may support the development of novel therapeutic options for infectious diseases and cancer.

## Introduction

Host cells activate the innate immune response in the presence of viruses and microorganisms by Pattern Recognition Receptors (PRRs). The PRRs recognize Pathogen Associated Molecular Patterns (PAMPs) and Damage Associated Molecular Patterns (DAMPs) aimed at pathogens clearance. Toll-like receptors (TLRs) and C‑type lectin receptors (CLRs) belong to the PRRs expressed by professional immune cells (e.g., macrophages and dendritic cells). In contrast, among PRRs ubiquitously expressed also in non-immune cells, there are NOD-like receptors (NLRs), RIG‑I‑like receptors (RLRs) and a group of intracellular DNA sensors such as cyclic GMP–AMP synthase (cGAS) and interferon-γ (IFNγ)-inducible protein 16 (IFI16)^1–4^. In the context of viral infection, exogenous RNA and DNA are recognized by specific cytosolic PRRs to activate pro-inflammatory cytokines and chemokines, in particular the type I IFNs, to counteract viral infection. In turn, secreted IFNs induce the transcription of IFN Stimulated Genes (ISG), which are crucial for the amplification of an anti-viral state^5^. STING is the principal actor of the cytosolic DNA sensing pathway. This pathway is upstream triggered upon the detection of cytosolic DNA by cGAS protein, which catalyzes the production of 2′3′ cyclic GMP–AMP (cGAMP) from ATP and GTP. This peculiar cyclic dinucleotide directly binds STING protein and induces a conformational change responsible for its activation. STING dimerizes and translocates from the endoplasmic reticulum to the Golgi apparatus where, upon a phosphorylation cascade, it activates IRF3 and NF-kB through TBK1. IRF3 and NF-kB transcriptionally activate type I interferon genes, tumor necrosis factor superfamily genes and proinflammatory cytokines. These soluble mediators of inflammation work as canonical autocrine and paracrine stimuli to alert neighboring cells of an imminent viral incursion. Interestingly, cGAMP is itself an unconventional immune transmitter. Indeed, cGAMP can be secreted as soluble or into extracellular vesicles or even be transferred to adjacent non-infected cells via gap junctions. This horizontal transfer elicits a rapid activation of STING and downstream signals in a cGAS-independent manner and conveys antiviral immunity in a transcription-independent way. These features make the STING pathway very effective in counteracting infections by DNA viruses^1,4,6^. While the STING pathway has been first depicted into antiviral immunity, nowadays it resulted involved in many systemic and organ-specific diseases^7^. The powerful immunity mediated by STING makes it an appealing target in cancer therapy, where many STING agonists are currently being investigated in tens of clinical trials (CT) (phase I to III).

More recently, an immune cells restricted function of STING has been also discovered in monocytes and macrophages where differentiation into classically or alternatively activated macrophages as well as antigen presentation seems to be affected by cGAS-STING axis via IRF3, IRF7 and STAT6^8,9^.

Human STING protein is encoded by *STING1* gene (also known as *TMEM173* or *MITA*). Interestingly, three different main allelic variants have been reported in human population known as wild-type R232, H232 with arginine to histidine substitution at position 232 (*rs1131769*), HAQ (R71H, G230A, R293Q) where R71H (*rs11554776*), G230A (*rs78233829*), and R293Q (*rs7380824*) are in linkage disequilibrium. Different distributions of these allelic variants have been reported in individuals of distinct ethnic populations suggesting that environmental pressure played a role in the selection of STING alleles. While the homozygous R232 allele is the dominant *STING1* genotype in Europeans, more than 50% of Americans have different genotypes. About 30% of Asians and 10% of Europeans are HAQ/HAQ, HAQ/H232, or H232/H232^10^. The functionality of the different allelic variants of STING has long been controversial. Despite the trend in scientific literature according to which different alleles have evolved differently to distinguish between endogenous cGAS-derived 2ʹ3ʹ-cGAMP from bacterial or metazoan cyclic di-nucleotides (CDNs; e.g., 3ʹ3ʹ-cGAMP), the use of different functional assays, different models, or recapitulation of human alleles into orthologue proteins (e.g., murine STING) complicated an unambiguous interpretation of results. Also, HEK293 were often used as a model to study STING, as they were considered as naturally STING-null, actually expressing a very low level of the protein, but still sufficient to trigger the IFNβ cascade in response to appropriate stimuli. Indeed, in a recent paper, we demonstrated that genetic knock-out of *STING1* by CRISPR-Cas9 produced a significant enhancement of *Herpes simplex* viral replication^11^. Finally, transient overexpression of a STING variant of interest by plasmid transfection acts, by itself, as a stimulus for endogenous STING protein, thus artificing interpretation of results. Many studies have been conducted with different cell lines naturally bearing different alleles, or with human-derived PBMCs, where modifying genes or STING-independent sensing of DNA (e.g., TLRs) could be misleading^12^. To further complicate these matters, the allele with histidine at position 232 was the first reference allele sequenced in the human genome project, so it has been long considered as a wild-type allele. For this reason, most of the crystal structures have been resolved on this H232 reference allele (NP_938023.1, denoted as hSTING^REF^). Only later it was shown that arginine at position 232 is the most common allele thus, nowadays it is considered as wild-type^13^. The HAQ and H232 alleles are the most controversial, since in Patel et al. HAQ and, to a less extent H232, were defined as loss-of-function alleles by characterizing response to stimuli in human patient-derived B cells^13,14^. While the mechanism of the hypomorphic alleles was not well defined it resulted, at least in part, associated with extremely low STING protein expression in HAQ/HAQ cells. A few months later, in a commentary to Patel et al., Sivick and colleagues reported on HAQ as a fully functional allele, using human PBMCs as model, although their study had a small sample size with unknown ethnic origins. The core of the discussion was the controversial use of B cells as an unsuitable model for STING studies and on the difficulties in recapitulating human alleles in mouse genes^15^. Few genome-wide association studies (GWAS) investigated the role of STING polymorphisms in pathological conditions, reporting that H232 and/or HAQ are associated to human cervical cancer and anergic response to poxviruses^16,17^. To overcome all these conflicting findings, companies screened their compounds across all genetic variants of STING^18,19^. Beyond the debated hypomorphic allelic variants of the *STING1* gene, both gain- and loss-of-Function (GOF, LOF) mutations have been described. GOF mutations were described as monogenic causes of severe pathological type I interferonopathy conditions known as STING-associated vascular disease (SAVI) with onset in infancy (i.e., V155M). On the contrary, loss of function mutations were often described in tumors, with the inactivation frequency directly proportional to the cancer stage. Indeed, under the selective pressure of the immune system, cancer cells inactivate STING protein by genetic mutations or epigenetic silencing as an escape mechanism to immune surveillance^20–23^. *STING1* status in tumors is a prognostic marker for different immunotherapeutic approaches. Deciphering the actual functionality of *STING1* alleles is thus essential also to drive the precision-medicine approach to oncologic patients’ cancer immunotherapy^24–26^. Considering the central role of STING in many biological processes (cancer, infectious disease, aging, etc.), it is of timely interest to define the functionality of these widespread alleles and to solve these controversies.

## Results

### THP-1 cell lines bearing STING1 allelic variants show different IFN and NF-kB responses upon administration of synthetic stimuli

**Figure 1:**
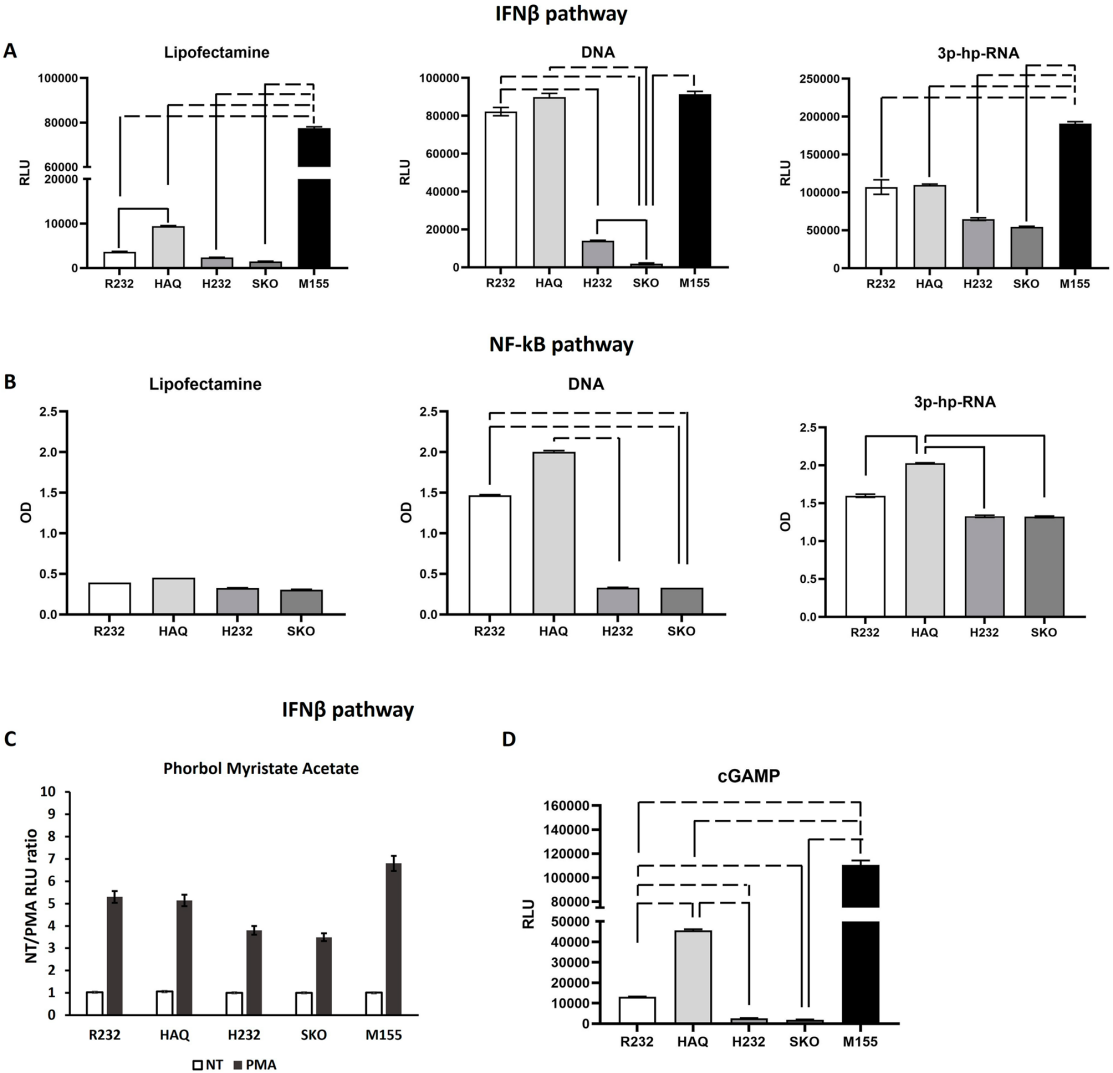
IFN and NF-kB pathway activities after different stimuli. THP-1 cell lines stably express a Secreted Luciferase (SLuc) and Secreted Embryonic Alkaline Phosphatase (SEAP) reporter genes respectively under the control of IFN and NF-kB responsive promoters. All cell lines were stimulated with a DNA stimulus or with a STING-unrelated stimulus (3p-hp-RNA). Luciferase (**A**) and SEAP (**B**) activities were assessed 24 h post stimulus from supernatants. Lipofectamine panels show the basal expression. Activation of IFNb pathway upon PMA stimulus was depicted in panel (**C**). Triggering of IFNb upon 2′3′cGAMP is shown in panel (**D**). The statistical analysis was performed by student’s *t*-test by multiple comparisons. Dashed lines indicate *p* < 0.005; solid line *p* < 0.05.

The contradictory results arising from the available scientific literature on *STING1* allelic variants are due to the differential backgrounds of the experimental models. So, we investigated the actual contribution of the genetic variants in STING-mediated innate immune activation in a unique syngeneic cellular model. Accordingly, we exploited THP-1 cells, a human monocytic cell line originated from a Japanese patient with acute monocytic leukemia^27^. While in Diner et al.^28^ it was found that THP-1 cells bear the HAQ allele of *STING1*, it is not clear whether these cells are homozygous or heterozygous for HAQ. We sequenced THP-1 *STING1 locus* and STING transcript respectively from genomic and cDNA by Sanger sequencing. We assessed that THP-1 are homozygous for HAQ allele (Supplementary Fig. S1). We implemented several cell derivatives that were knock-out (Fig. S1) and knock-in for the *STING1* allelic variants of interest (R232, HAQ, H232). The bona fide of knock-in was validated by Sanger sequencing of cDNA (Supplementary Fig. S1). The knock-in of alleles in the *STING1 locus* allows to preserve its genetic and epigenetic regulations. GOF V155M mutation was also implemented as a positive control model in the current study. Beyond genetic variations in *STING1* locus, all these cell lines stably express a secreted luciferase (SLuc) and Secreted fetal alkaline phosphatase (SEAP) reporter genes respectively, under the control of IFN and NF-kB responsive promoters. We stimulated all these genetic background matched cell lines with a synthetic DNA stimulus to trigger the activation of the cGAS-STING axis. Luciferase and SEAP activities were assessed 24 h post stimulus from supernatants as reporter for IRF3-IFNs and NF-kB activation (Fig. 1A). THP-1 R232 cell line, considered as WT allele, showed a functional IFN pathway after DNA stimulus. Surprisingly, the IFN pathway was activated in THP-1 HAQ cell line by DNA stimulus in a similar fashion to THP-1 bearing R232 allele, while THP-1 H232 cell line showed an impaired IFN pathway, whereby defined as a Loss of Function allele. As expected, THP-1 STING KO (SKO) cell line didn’t activate IFN pathway. We also evaluated NF-kB activity after 24 h post stimulation through SEAP assay (Fig. 1B). Also in this case, THP-1 R232 and HAQ cell lines showed an active NF-kB pathway similar to each other. Still, THP-1 H232 and SKO cell lines showed an impaired NF-kB signaling. As expected, THP-1 bearing the M155 allele resulted constitutively and stimulus-independent activated. As a control, a nucleic acid based, STING-independent RNA stimulus (3p-hp-RNA) was used to verify the functionality of the model, demonstrating that all cell lines bearing different alleles as well as STING KO cells were correctly activated by a STING-unrelated stimulus. The slightly dampened activation by 3p-hp-RNA of H232 and STING KO cells compared to R232 and HAQ was expected as STING is at least in part also involved in RNA sensing^29,30^.

Phorbol Myristate Acetate (PMA) was used as second control as nucleic acid unrelated stimulus showing no significant differences between alleles in IFNb activation (Fig. 1C).

**Figure 2:**
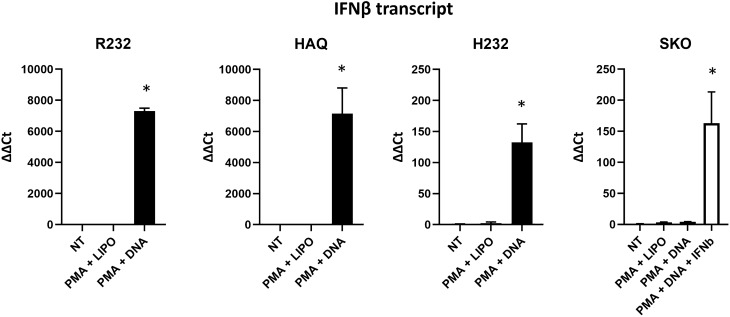
IFNβ transcriptional activation in differentiated macrophages. THP-1 cell lines were treated with phorbol myristate acetate (PMA) alone or with PMA in combination with a DNA stimulus. After 18 h the transcriptional activation of endogenous IFNβ was evaluated into differentiated macrophages. Functional rescue was performed using supplementing recombinant IFNβ in the supernatant of STING KO cells (white bar). The statistical analysis was performed by student’s *t* test using NT of each allele as reference.

Monocytic derivation of THP-1 cells makes them able to sense cytoplasmic DNA also by TLRs that give a huge contribution to nucleic acids response. For this reason, we investigated IFN-I response using the selective STING activator, 2′3′ cGAMP. As shown in Fig. 1D, these results confirmed the functionality of R232 and HAQ alleles while H232 e SKO cell lines resulted unable to activate the pathway. This proved that the obtained evidence was actually associated to the functionality of STING. Nf-Kb pathway followed the same trend as IFN-I even if as administered at sub-active concentration, 2′3′ cGAMP exerted less evident differences between alleles (data not shown). Additionally, we evaluated the actual transcriptional activation of endogenous IFNβ, a direct target of STING pathway into differentiated macrophages. THP-1 cells were thus treated with PMA alone, or in combination with a DNA stimulus. Combination of PMA and DNA stimulus strongly activated IFNβ in R232 and HAQ cells. On the contrary, H232 only barely activated IFNβ with an intermediate strength compared to STING KO cells, thus corroborating its hypomorphic function (Fig. 2). We also dosed IFNβ protein levels in R232 cellular supernatant by ELISA, showing a range between 0.5 and 1.5 ng/ml (data not shown). We thus supplemented the supernatant of STING KO cells with 1 ng/ml recombinant IFNβ to verify the contribution of IFNβ feedback on its own transcription. This supplementation only partially rescued transcription via IFNβ receptor signaling, demonstrating the relevance of the STING-IRF3 axis to amplify antiviral immunity (Fig. 2). IFNβ activation in response to PMA and DNA stimuli was also assessed in M155 cells, where IFNβ was up-regulated upon DNA stimulus, despite the high constitutive basal expression (Fig. S2).

### STING1 allelic variants affect the sensing of HSV-1

**Figure 3:**
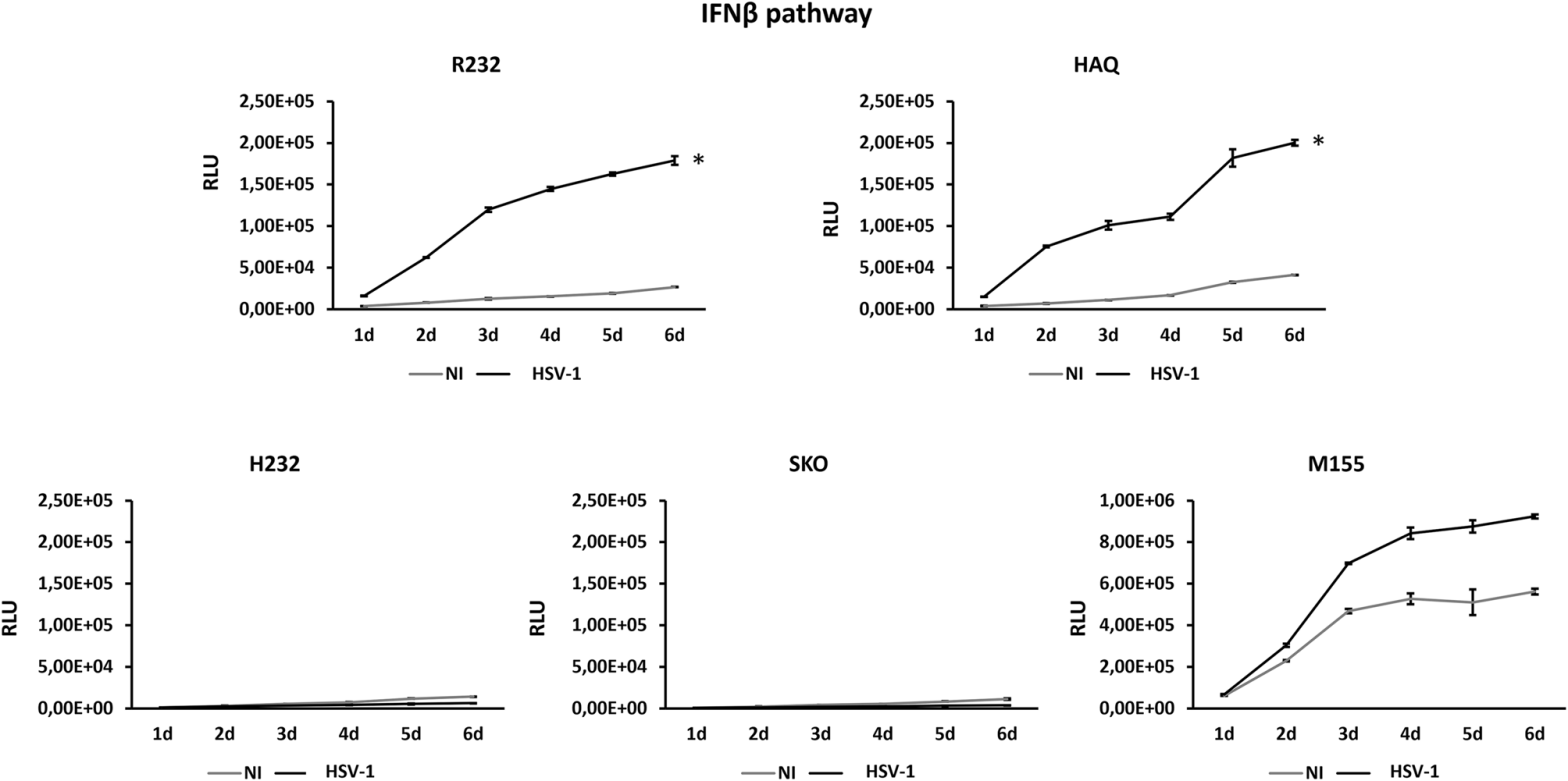
IFN pathway activity after HSV-1 infection. THP-1 cell lines stably express a secreted luciferase (SLuc) reporter gene under the control of IFN responsive promoter. All cell lines were infected at MOI 0.1 pfu/cell with HSV-1. The media were collected from day 1 to day 6 after infection and luciferase activity was assessed. NI lines (grey lines) show the basal expression of the cell lines. The statistical analysis was performed by student’s t-test using NI of each allele as reference.

Having confirmed the different functionality of STING alleles, we investigated whether and how these variants could affect the susceptibility to viral infections. To do this, we exploited *Herpes simplex* virus Type 1, a DNA virus, to infect THP-1 cell lines and study the downstream effects derived from the infection considering the contribution of allelic variants. We infected cells at low multiplicity of infection, MOI 0.1 pfu/cell (1 pfu over 10 cells) to allow viral cycling and replication and we collected the supernatants in a time-course experiment (Fig. 3). After a viral infection, THP-1 cell lines bearing R232 and HAQ alleles showed an active IFN signaling even at early time point (day 1_18h) that amplifies during the time. On the contrary, THP-1 H232 and SKO cell lines were completely defective in triggering IFN pathway. THP-1 M155 cell line confirmed its GOF behavior as showed comparing not infected and infected samples (Fig. 3).

**Figure 4:**
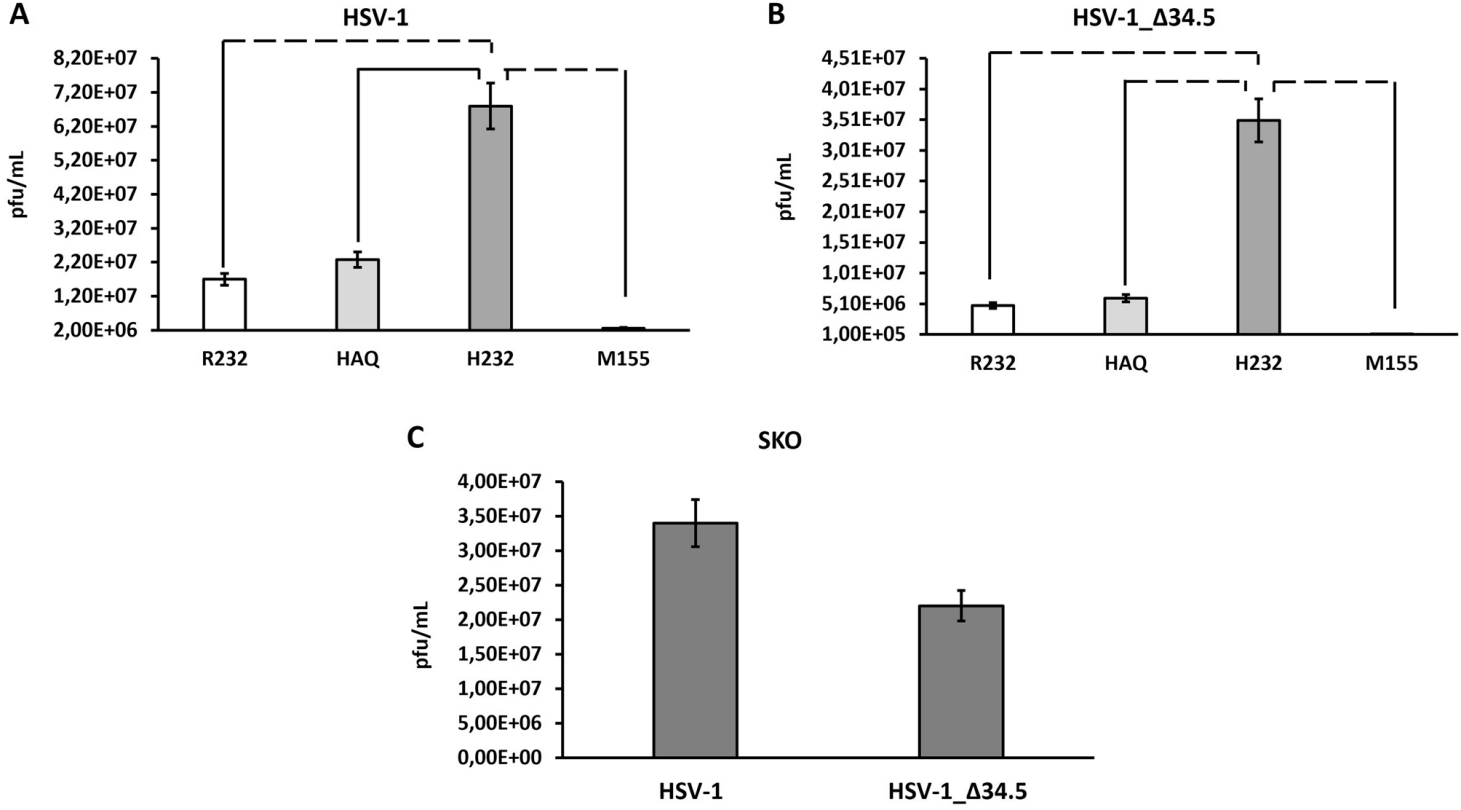
Viral replication in THP-1 cell lines. Analysis of HSV-1 (**A**) and HSV-1_Δ34.5 (**B**) viral titers obtained in R232, HAQ, H232 and M155 THP-1 cell lines infected with MOI 0.1 pfu/cell. Plaque assay was performed in a cell bulk as biological replicate. (**C**) Shows the viral replication of both viruses in STING KO cell line. The statistical analysis was performed by student’s *t*. Dashed lines indicate *p* < 0.005; solid line *p* < 0.05.

We thus assessed viral replication in cell lysate to evaluate the ability of different STING variants to interfere with viral propagation. As expected, the replication of HSV-1 resulted significantly improved (threefold) in terms of infectious viral particles production in H232 cells compared to HAQ and R232 ones (Fig. 4A). Many viruses evolved effective mechanisms to avoid detection by innate immune sensors. HSV-1 evolved into γ34.5 protein the accessory function to inhibit STING to escape its DNA recognition^31^. We thus expected that by inhibiting STING, wild type HSV-1 could in this way flatten the differences between H232 and R232 STING alleles. To strengthen the STING allele-dependency of these phenotypes, we generated an HSV-1 deleted in γ34.5 *loci* (HSV-1_Δ34.5), as the clinical approved oncolytic T-VEC virus^32^. The differences in viral replication between H232 and R232 or HAQ were exacerbated even more in the absence of 34.5 protein (one order of magnitude) (Fig. 4B), thus proving the allelic variant dependency of the reported phenomena. As expected, the replication of wild type HSV-1 and Δ34.5 viruses resulted similar in *STING1* knock-out cells, where the contribution of STING is completely abolished (Fig. 4C). Finally, both wild-type HSV-1 and HSV-1_Δ34.5 viruses lost their replication capacity in M155 cell line where STING cannot be counteracted by the viral proteins, due to the constitutive activation of STING (Fig. 4A,B).

**Figure 5:**
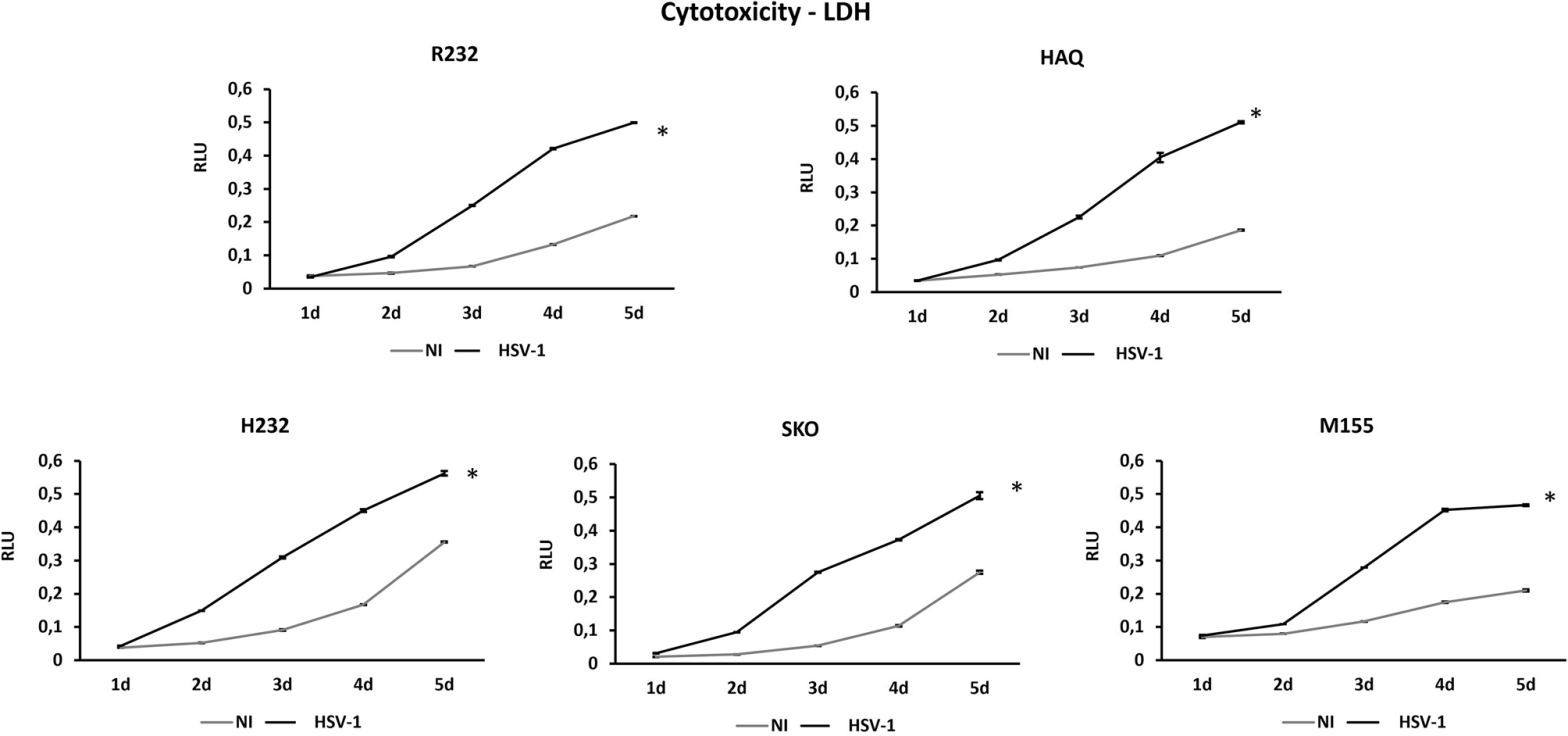
Cytotoxicity of HSV-1 in THP-1 cell lines. The lytic activity of HSV-1 was evaluated by extracellular LDH (lactate dehydrogenase) release in cell supernatants over the time course of infection (day 1 to day 5). All the cell lines were infected at MOI 0.1 PFU/cell. All the infections were performed as biological replicates. NI lines (grey lines) show the LDH basal expression of the cell lines. The statistical analysis was performed by student’s *t* test using NI of each allele as reference.

The results obtained with wild-type and with HSV-1_Δ34.5 herpesviruses are in line with those obtained with synthetic stimuli, confirming the loss of functionality of the H232 allele of STING, and the full competence of HAQ and R232 variants. Assumed the contribution of STING alleles in viral replication, we investigated their involvement in the cytotoxicity potential induced after viral infection. We infected all five cell lines with wild-type HSV-1 at MOI 0.1 pfu/cell and assessed cell viability over five days. Surprisingly, the cytotoxicity assessed as LDH release did not reflect the activation of STING pathway, where all the cell lines (STING KO, STING R232, H232, HAQ) succumbed with a similar trend (Fig. 5). We also assessed the actual percentage of live cells but, also in this case, the results were consistent with the LDH release assays (Fig. S3). The absence of direct proportionality between viral replication and cell killing spurred us to hypothesize that cell death was not induced by the lytic-cycle of HSV-1. The ultimate expression of this speculation is evident in M155 cells where, although replication of HSV-1 was completely abrogated (Fig. 4), the cell death followed the same trend as in STING knock-out or H232, where the highest viral load was achieved. As THP-1 cells express high levels of TLRs, known to mediate downstream caspase activation and cell death, we moved into a non-immune cellular system^33^.

### Hypomorphic STING alleles affect susceptibility and IFN-I activation in response to DNA viral infection

**Figure 6:**
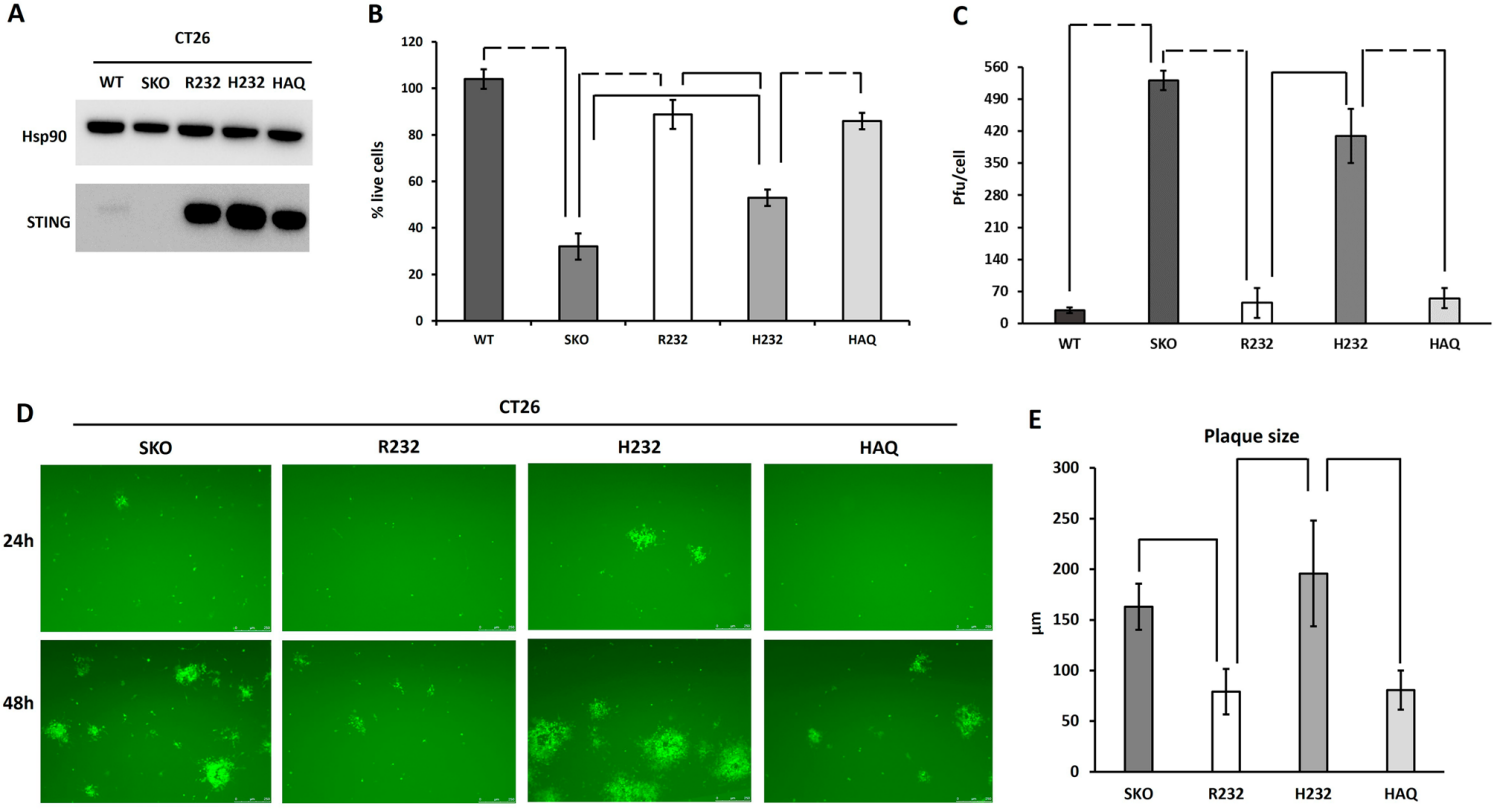
Characterization and susceptibility of genetically modified CT26 cell lines. (**A**) Western blot analysis of STING protein in murine STING CT26 (WT), CT26 STING KO and in human STING CT26 KI cell lines (R232, H232, HAQ). Hsp90 protein was used as standard. (**B**) Cell viability in CT26 WT, CT26 SKO and CT26 STING-KI cell lines after HSV-1 infection at MOI 0.1 pfu/cell. The percentage of live cells was evaluated after 48 h post infection and was obtained by counting live cells on the non-infected count. The statistical analysis was performed by student’s *t* test using CT26 WT as reference. (**C**) The titre of virus in cell lysate was performed by plaque assay. (**D**) The spread of eGFP-encoding HSV-1 was evaluated by fluorescence microscopy in CT26 STING-KI cell lines after 48 h post infection. Panel E show the difference in plaque size between CT26 SKO and STING-KI cell lines in terms of um referred to panel **D**. The statistical analysis was performed by student’s *t* test. Dashed lines indicate *p* < 0.005; solid line *p* < 0.05.

To avoid the interference caused by the presence of TLRs, we generated a non-immune cell model knock-out for *Sting1* (STING KO) based on the CT26 cell line. Starting from this background, we stably restored the human allelic variants of interest R232, H232 and HAQ. Stable expression of STING variants was assessed over 20 cell passages (Fig. 6A and Supp. Fig. S4). Expression over 20 passages demonstrated that different alleles do not affect protein stability nor abundance.

We infected the above cell lines with an HSV-1 virus derivative encoding eGFP into an intergenic, non-deleterious *locus* at MOI 0.1 pfu/cell. We evaluated cell viability demonstrating that R232 and HAQ bearing cells were almost resistant to viral infection; on the contrary, H232 cells succumbed similarly to *Sting1*KO cells (Fig. 6B). Wild-type CT26 cells expressing the endogenous STING protein were used as control. Here, the cell viability resulted comparable to the one assessed in R232 and HAQ cells. These data reflect the differences in STING alleles functionality; in particular, the cell lines in which STING works properly (endogenous wild type, R232 and HAQ) are less permissive to viral replication and consequently more viable than cells in which STING function was reduced or absent (H232 and SKO, respectively). Also, viral yield was assessed in cell lysate finding a linear correlation between cytotoxicity and viral replication (Fig. 6C).

**Figure 7:**
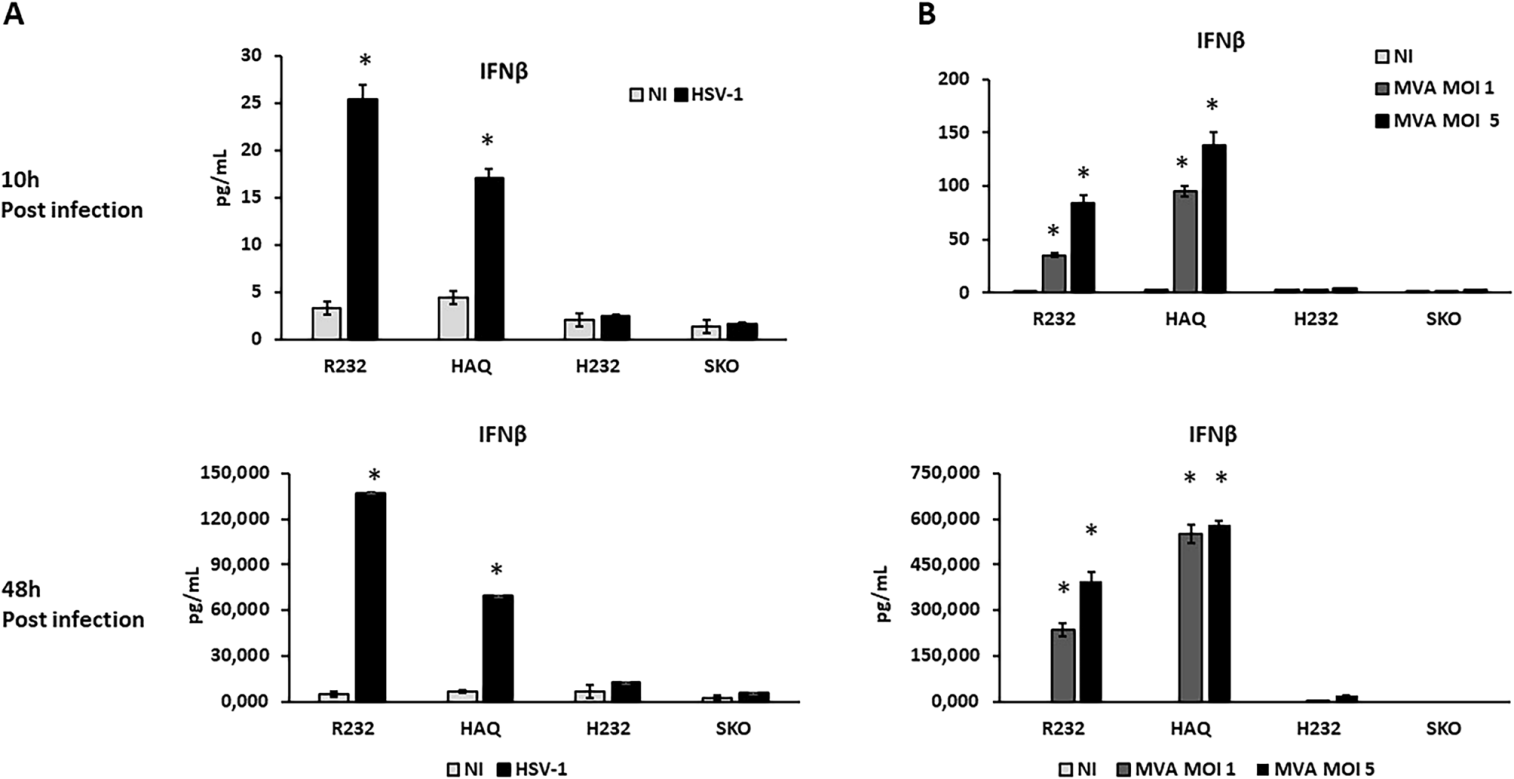
IFNβ secretion in CT26 cell lines induced by viral infection. (**A**) CT26 cell derivatives (R232, HAQ, H232 and SKO) were infected at MOI 10 pfu/cell with wild type HSV-1 and the supernatants were collected 10 h and 48 h post infection to dose interferon beta. The statistical analysis was performed by student’s *t* test using not infected cells as reference. (**B**) The same experiment was performed using a different DNA-based virus, MVA at MOI 1 and 5. The statistical analysis was performed by student’s *t* test using NI of each allele as reference.

As already described, beyond the IFNβ secretion, cGAS-STING axis can horizontally transfer warning signals to bystanders cells via cell-to-cell passage of cGAMP through gap junctions. Thanks to the eGFP encoded into our engineered HSV-1 genome, we assessed viral spread by infecting a cell monolayer with 1 pfu over 100 cells (0.01 MOI). We evaluated the spread as GFP-positive plaques at 24 h and 48 h post infection. CT26 Sting1 KO and H232 allowed rapid viral spread compared to cells bearing R232 and HAQ alleles (Fig. 6D). Viral spread in wild-type CT26 expressing endogenous STING protein was also assessed, resulting similar to the values observed for R232 and HAQ variants (Fig. S5). The differences in plaque size (µm) were statistically significant between H232 and R232 cell lines (Fig. 6E). On the contrary, H232 and *Sting1* KO cells allowed an overlapping spread of viral particles. These results show how the presence of STING interferes with viral spread and, in turn, with the cytotoxicity induced by the viral infection. We also characterized the downstream STING pathway by investigating IFNβ secretion induced by viral infection. We infected CT26 cell derivatives (H232, HAQ, R232 and *Sting1*KO) with-wild type HSV-1 and collected the supernatants 10 h post infection and 48 h post infection to assess interferon beta secretion. As expected, induction of IFNβ 48 h upon infection resulted very high in R232 and HAQ bearing cells, while resulting not statistically significant in CT26 *Sting1*KO and H232 bearing cells (Fig. 7A). Although with less pronounced differences, alto 10 h post infection IFNb secretion was significantly high in R232 and HAQ cells while not-detected in Sting KO and H232 cells (Fig. 7A).

This result further confirmed the essential, non-redundant role of STING in recognizing HSV-1 virus and the hypomorphic (near-null) activity of H232. Beyond HSV-1, we further aimed to expand the characterization to additional DNA viruses selecting Modified Vaccinia Ankara (MVA), an attenuated vaccinia virus widely considered as the vaccinia virus strain of choice for clinical investigation in vaccinology and gene therapy because of its high safety profile^34^. As for HSV-1, also MVA failed to activate STING H232, as assessed by IFNβ secretion close to the detection limit. On the contrary, HAQ and R232 efficiently promoted secretion of IFNβ upon MVA infection, up to hundreds of pg/ml (Fig. 7). While with HSV, the differences were evident, especially at 2 days, with MVA, IFNb secretion reached the hundreds pf picograms even 10 h post infection. CT26 cells expressing endogenous STING protein were also infected with HSV-1 and MVA showing IFNβ secretion in a similar fashion to HAQ and R232 cells (Fig. S5).

To understand if the main final output of STING pathway was affected, we exploited nuclear translocation of IRF3 as a faithful indicator of its phosphorylation and related pathway activation^35^.

**Figure 8:**
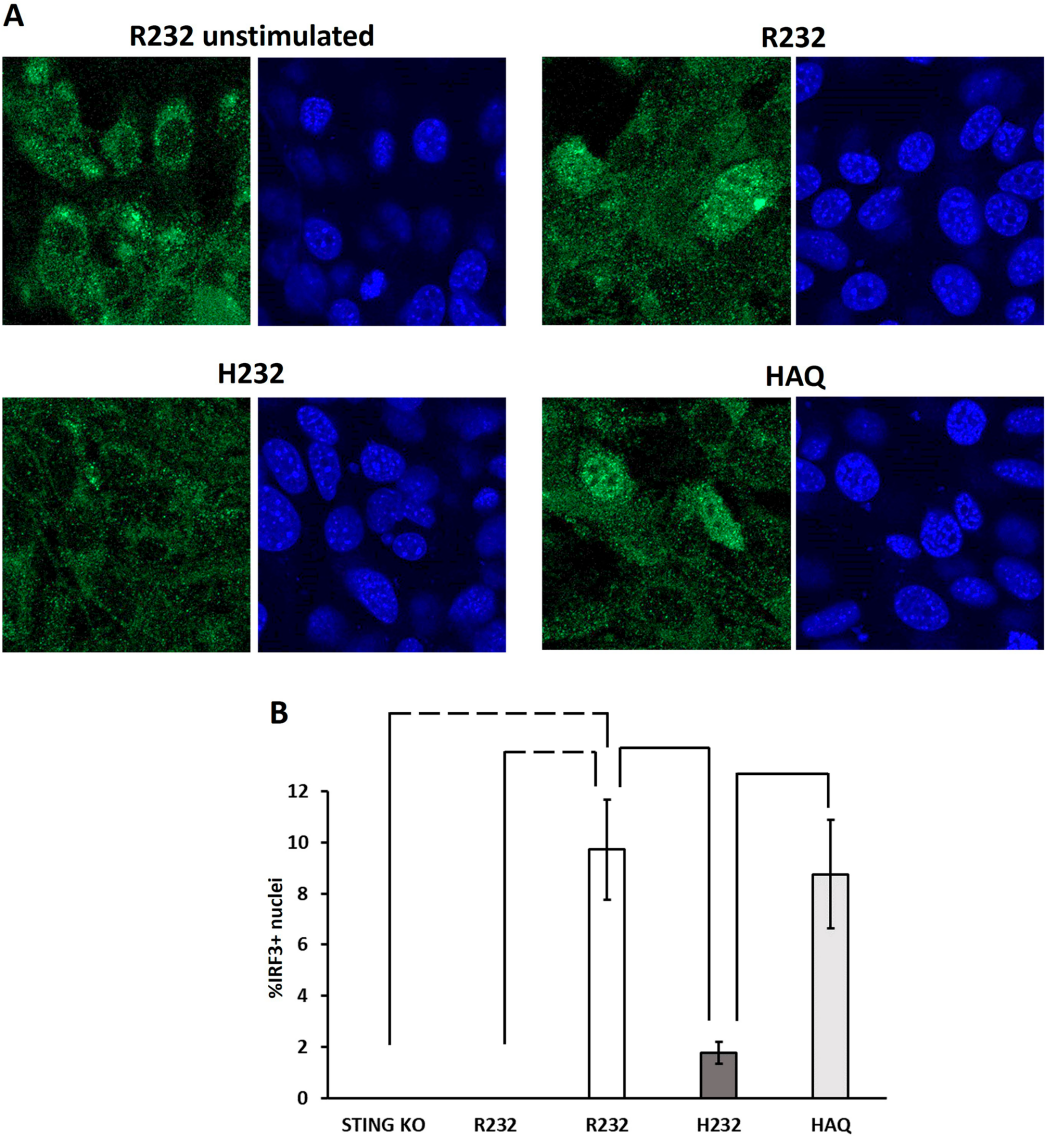
Nuclear translocation of IRF3 in CT26 cell lines induced by DNA stimulus. (**A**) CT26 cell derivatives (R232, HAQ, H232) were stimulated with DNA (1 µg/ml). Cells were fixed 8 h after stimulation and nuclear translocation was assessed by IF. R232 non-stimulated cells were used as negative control. (**B**) Unbiased count of nuclear IRF3 + cells by DAPI/IRF3 colocalization. The statistical analysis was performed by student’s *t* test. Dashed lines indicate *p* < 0.005; solid line *p* < 0.05.

Nuclear translocation of IRF3 was evaluated as direct readout of STING activity by immunofluorescence in R232 unstimulated and in DNA-stimulated cells (R232, H232, HAQ). Figure 8A shows representative images of cells where IRF3 (green) results translocated in about 10% of cells in R232 and HAQ cell lines, whereas only an approximatively 2% of H232 cells show nuclear IRF3. In panel B of Fig. 8, an unsupervised count of DAPI/IRF3 colocalization is depicted. STING KO stimulated cells and different images are shown in supplementary Fig. S6.

### STING1 allelic variants contribute to macrophage differentiation and antigen presentation in THP-1 monocytes cell lines

**Figure 9:**
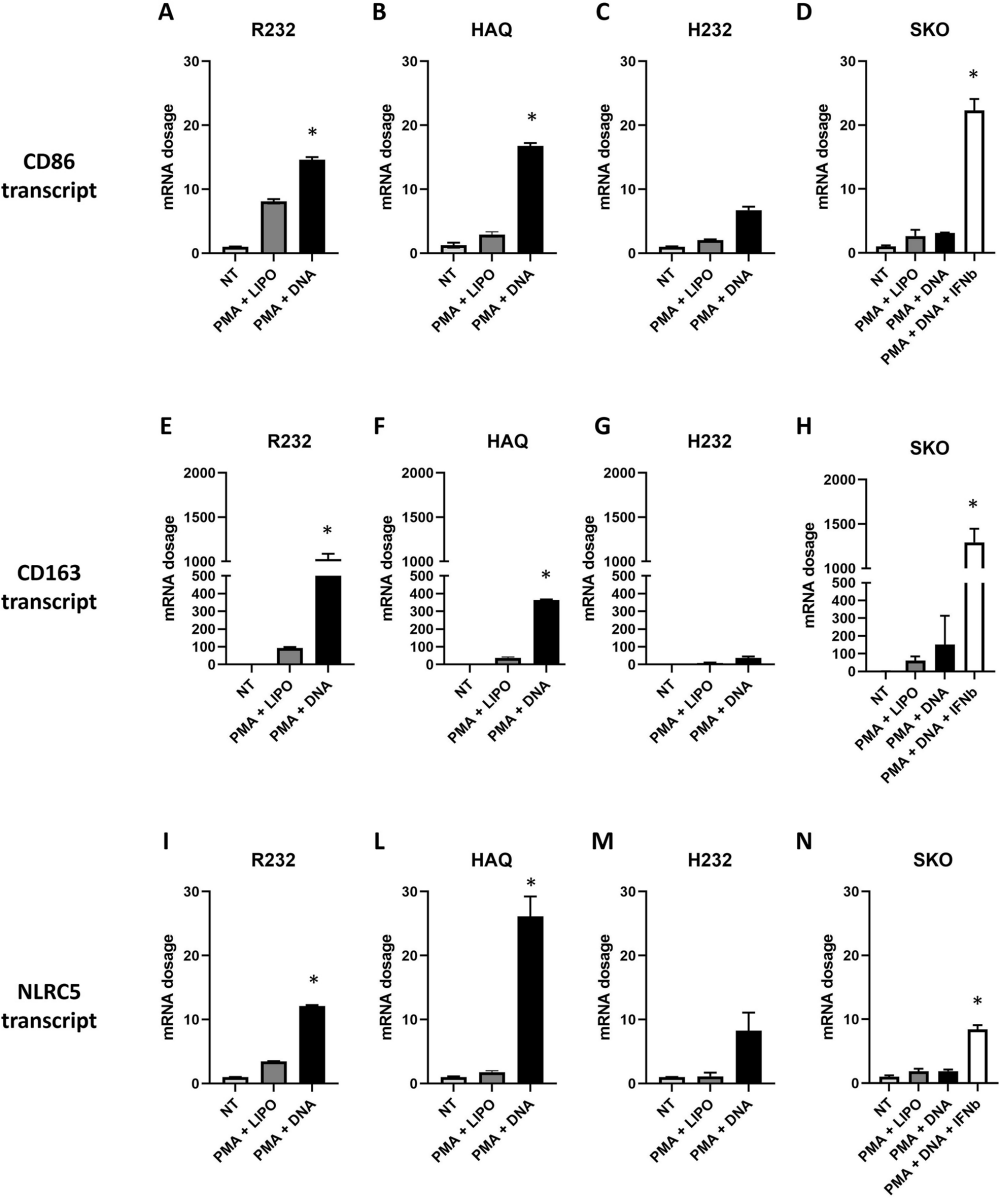
Transcriptional activation of differentiation and activation markers into differentiated macrophages. THP-1 R232, HAQ, H232 and STING KO cell lines were treated with phorbol myristate acetate (PMA) alone or with PMA in combination with a DNA stimulus. After 18 h total RNA was extracted to evaluate: M1 marker CD86 (**A**, **B**, **C**, **D**); M2 marker CD163 (**E**, **F**, **G**, **H**); NLRC5 (MHC class I trans activator) (**I**, **J**, **K**, **L**). A “passive” rescue was performed in STING KO cells by supplementing recombinant IFNβ in the supernatant (**D**, **H**, **L**). The statistical analysis was performed by student’s *t* test using NT of each allele as reference.

It is well known the stimulating role of STING protein on the immune system. In particular, STING can stimulate different pathways leading to the expression of plenty of genes and cytokines involved in monocytes differentiation^36^. As expected, upon differentiation of THP-1 cells with PMA, cells become adherent but still rounded as M0 macrophages. We noticed that the addition of STING stimulus induced a dramatic change in cellular morphology in R232 cells that acquired stellar shapes typical to differentiated macrophages. On the contrary, STING KO THP-1 cells resulted still adherent and rounded after nucleic acids stimulation (Fig. S7). Based on this observation, we investigated how the *STING1*gene, and its allelic variants, could influence monocyte differentiation. For this purpose, THP-1 cells were treated with PMA and then transfected with DNA to activate STING pathway. Eighteen hours post stimulation, we evaluated the expression of macrophage polarization markers. In particular, we analyzed the expression of CD86 as a marker of M1 polarization (Fig. 9A–D) and CD163 as a M2 marker of polarization (Fig. 9E–H). The expression levels of M1 and M2 markers in SKO and H232 cells (Fig. 9C,D,G,H) were lower, in comparison to R232 and HAQ cells (Fig. 9A,B,E,F). While divergent M1 or M2 polarization is usually expected, the co-regulation of both M1 and M2 markers is usually associated to strong inflammatory stimuli^37^. It was previously reported that CD86 and CD163 are IFN-stimulated genes (ISGs) as transcriptionally activated downstream of the IFN-I receptor (IFNAR) signaling by Interferon-sensitive response element (ISRE)^38^. We thus supplemented STING KO cell media with human recombinant IFNβ showing how the extent of transcriptional activation of CD86 and CD163 were rescued as in R232 cells (Fig. 9D,H). We supposed that STING and its functionality also influence the antigen presentation process. We thus evaluated expression levels of NLRC5 (CITA), a master regulator of class I antigen presentation in all STING cell derivatives upon DNA stimulation. As opposed to CD86 and CD163, NLRC5 is a potential direct target of STING cascade, as suggested by the presence of IRF3 responsive elements (interferon stimulation regulatory element (ISRE)) in its promoter^39^. In agreement with our hypothesis, the functional integrity of STING protein (R232 and HAQ) was related to a higher expression of class I trans activator NLRC5 (Fig. 9I,J), while defective activation (STING KO, H232) led to very lower expression (Fig. 9K,L). Also in this case, we treated STING KO cells with recombinant IFNβ to understand how NLRC5 is closely STING-dependent. As expected, the ectopic supplementation of recombinant IFNβ in THP-1 STING KO medium only partially restored NLRC5 expression. Activation of CD86, CD163 and NLRC5 was also assessed upon PMA or PMA with DNA stimulus into M155 cells. Here, although the basal expression was high, due to constitutive activation of STING, we were able to appreciate a further transcriptional enhancement of CD86, CD163, NLRC5 upon DNA stimulus (Fig. S8).

## Discussion

Since STING was first discovered about a decade ago as an antiviral protein, continual research revealed its intriguing involvement in non-infectious diseases. The first key discovery regarded the ubiquitous expression of STING and of its upstream activator cGAS in cells from different tissues and organs. STING ubiquitous expression spurred scientists to investigate its function in many fields including, but not limited to metabolic disorders, cancer, autoimmune diseases, neurological disorders, cardiovascular diseases and, more recently, aging^40^. Depending on the field of interest, activation or inhibition of STING could be desirable, according to exemplified paradigms, respectively, in cancer and autoimmune diseases^41^. Despite STING appearing as a canonical actor in a cell pathway, early, conflicting evidence arose. First, an IFN-independent STING function was discovered, underlining that STING acts differentially in a cell-dependent context (i.e., lymphocytes or monocytes)^42^. Second, despite human *STING1* has conserved orthologues in vertebrates, small differences in the C-terminal tail can push the pathway towards IRF-3 (in human, non-human primates and mouse) or NF-κB (fish)^43,44^. Third, at least three main human allelic variants of STING have been identified with different distributions in distinct ethnic populations (R232, H232, HAQ). Due to studies conducted in different species (e.g., mouse, zebrafish), or in human cell derivatives with different genetic backgrounds, the functionality of these alleles is still debated and ambiguous^45^. The broad spectrum of exploitation of STING pathway knowledge generated a great enthusiasm encouraging companies to rapidly bring STING agonists to the clinic but facing continual premature termination or suspension of clinical trials. This is the striking case of the STING agonist DMXAA (Vadimezan), implemented for cancer therapy, that showed encouraging preclinical results but failed in phase III clinical trial where showed no clinical benefits (NCT00662597). Only later, further research revealed that DMXAA efficiently activates murine but not human STING. The second-generation STING agonist ADU-S100 (also referred to as MIW815) developed by Aduro Biotech in collaboration with Novartis also faced with clinical trial discontinuation due to lackluster clinical data. While ADU-S100 was shown to activate all known human and mouse STING alleles, it is well known that STING is often inhibited in cancer by mutations or by epigenetic silencing^20–23^. As ADU-S100 was administered intratumorally, the lack of inclusion/exclusion criteria based on STING status in tumours could have affected the results^46^. Indeed, a tumour-intrinsic activity of STING has been revealed as essential to mediate immunogenic cancer cell death and antitumor immunity^47,48^. Also, Merck Sharp and Dohme Corp underwent phase I clinical trials with STING agonist MK-1454 administered intratumorally in combination with pembrolizumab, showing encouraging efficacy and an acceptable safety profile^49^. More recent clinical trials investigating next-generation STING agonists developed by Bristol-Myers Squibb, GlaxoSmithKline and others (BMS-986301, E7766, GSK3745417) include an arm where the compound is administered via IV injection^49,50^. The enthusiasm for implementing therapies targeting STING pathway became a double-edged sword and made it necessary to take a step back to basic science and preclinical testing. While genome-wide association studies suggested an impact of STING allelic variants on its functionality, this aspect has never been systematically approached and evaluated^10^. Here, for the first time, we assessed the functionality of R232, H232 and HAQ STING alleles in comparison to a STING knock-out model in two different genetic background matched systems. In both monocytic cell line model (THP-1) and non-immune cell line (CT26) we demonstrated that R232 and HAQ are equally functional, whereas H232 variant is severely impaired in the canonical STING1 function. This was the actual case, following both synthetic stimuli (DNA and 2′3′cGAMP) and viral infections. Our systematical work that makes a point of STING alleles functionality may reveal great potential for the clinical translation of STING-targeting drugs. This is the case of both small molecules STING agonists or inhibitors, as well as viral vector-based therapies (e.g., gene therapy and oncolytic virotherapy). Here, we tested two clinically relevant viruses (HSV-1 and MVA), both used for gene therapy, oncolytic virotherapy and as vaccine shuttle^34^. In our previous work, as well as in different studies from Deng laboratory, it was demonstrated that tumor-intrinsic functionality of STING is essential to mediate oncolytic cancer immunotherapy^47,51,52^. While previously, the only restriction was thought to be associated with tumor-resident STING inactivation occurring under selective pressure of immunosurveillance, now, with this work we propose that also STING allelic variants could affect tumor-intrinsic antitumor immunity. Beyond this tumor-intrinsic function, STING plays a fundamental role also in non-tumor cells where STING Dependent Adjuvants (STAVs) (DNA of dying tumor cells, cGAMP, oncolytic viral DNA) can activate antigen presenting cells by phagocytosis^53^ as well as by transfer via gap junctions to tumor-associated dendritic cells (DCs) and macrophages, which respond by producing type I interferons in situ^54^. Such *cis*- and *trans*- activities of STING strongly underline how understanding the functionality of these alleles is of great interest for precision medicine approaches in oncology field. Not only we confirmed a previously reported involvement of STING in antigen presentation process, but also shed light on how hypomorphic STING alleles may affect APC maturation and antigen presentation.

**Figure 10:**
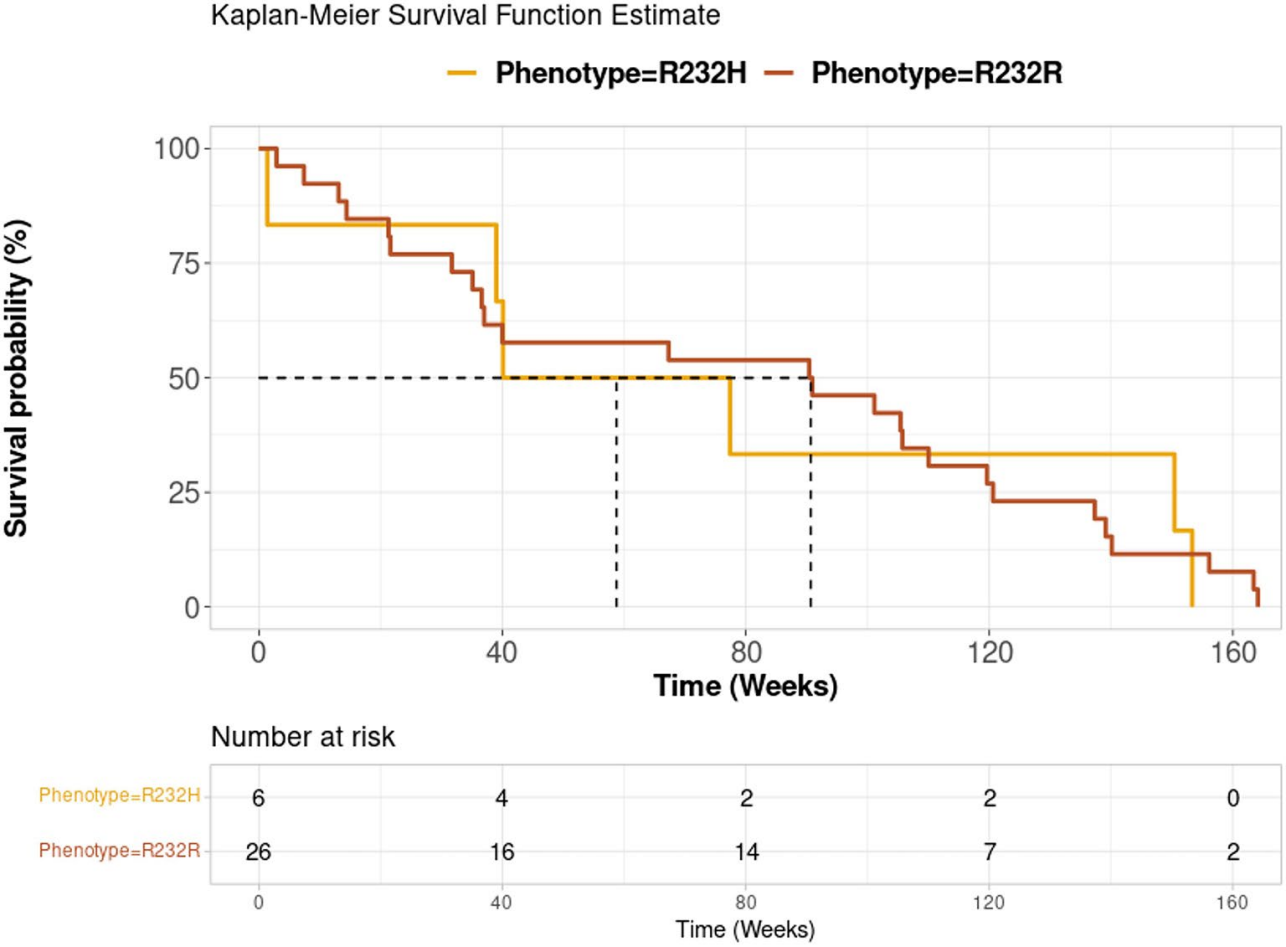
Kaplan Meier survival function estimate in R232/R232 vs H232/R232 melanoma patients treated with Nivolumab. A cohort of melanoma patients treated with nivolumab as first line was assessed for *STING1* allele. R232 homozygous and H232/R232 heterozygous patients were respectively showed as yellow and red curves. The table indicate the number of patients at given week.

Besides those roles inherent to DNA sensing, the functionality of STING in both tumor and non-tumor compartments may play roles the in elicitation of anti-tumor immunity in response to checkpoint inhibition (i.e., PD-1 and CTLA-4), as a possible consequence to sensing of cytoplasmic DNA generated by genotoxic stresses or chromosome instability^48^. This STING-mediated antitumor immunity is essential for spontaneous and therapy-induced antitumor T cell responses, putting STING as a link between innate and adaptive tumour immunity; under this rationale, functional characterization of STING alleles may reveal of relevance in precision medicine approaches^55^. Accordingly, we performed a look-up analysis to determine the impact of the presence of H232 (*rs1131769*) in a cohort of 32 melanoma patients treated as first line with PD-1 blockade Opdivo, (nivolumab). In concordance with the previously reported frequency in the world population, the arginine in the position 232 is encoded in homozygosis in the 75% of patients, while the minor allele encoding histidine is represented in the remaining 25% of patients. Unfortunately, given the small sample size, we were not able to identify patients homozygous for the H232 allele. However, we compared by Kaplan Meier survival analysis the differences between homozygous R232/R232 (26) *vs* heterozygous R232/H232 (6) patients. Although in heterozygous patients the presence of R232 can compensate for the hypomorphic H232 allele, we highlighted a nearly statistically significant difference in the overall survival of the patients carrying R232 in the homozygous combination with increased survival, compared to the heterozygous H232/R232 patients. Beyond the overall survival, Kaplan Meier curves showed a trend of nearly statistically significant divergence at 90 versus 50 weeks (Fig. 10), deserving further investigation. Despite this, we showed a nearly statistical significance in overall survival, suggesting that important differences would be revealed if H232/H232 patients were included into analysis. Although results were not statistically significant, we decided to share this data with scientific community as the obtained trend suggests that the observed biological effect could be relevant to identify strong evidence of a genetic basis for stratification of patients, according to *STING1 genotype*. Unfortunately, we are underpowered to detect it, so that we reasoned that these data could represent an assist for researchers interested in *STING1* analysis and collecting clinical data spanning from cancer therapy with checkpoint inhibitors, oncolytic viruses (e.g., TVEC) and other immunotherapeutic regimens, to gene therapy with DNA viruses, infectious and autoimmune diseases^56–64^.

Many open questions remain to be elucidated, including the potential protective effect by the H232 allele from autoimmune, cardiovascular or aging-related diseases, where proinflammatory activity of STING may accelerate degenerative processes, as reported for the less common *loss-of-function* R293Q allele^65,66^. An additional limitation of this work faces the lack of deep characterization of the molecular mechanisms underlying the activation cascade downstream STING alleles. As known, STING cascade is very complex and consists of multistep process including but not limited to binding of cGAMP, extensive conformational changes that also foster the oligomerization by disulfide bridges together with ER-to-Golgi translocation. Here, through a conserved motif, the C-terminal tail of STING recruits TBK1 that auto phosphorylates itself. pTBK1, in turn, further activate STING by phosphorylating serine in position 366 in the C-terminal tail. This conformation finally allows the STING–TBK1 complex to recruit and activate (by phosphorylation) IRF3. pIRF3 can dimerize and can translocate to nucleus to activate target genes. This work only in part address to the mechanism of hypomorphic allele H232, by looking at the last step by nuclear IRF3 translocation of indirect readout of phosphorylation cascade, but represent a solid bulk data for future investigations. . While these data are in countertrend to the controversial report that points HAQ as null allele^10,14^, it is possible to read the same trend between the lines in several papers^15,67^. Last, but not least, it will be attractive to understand how such a *loss-of-function* allele (H232) gained fitness in the long-term evolution. While natural selection has been proposed for the functional HAQ and AQ alleles during the out-of-Africa migration^68^, it can be hypothesized that the H232 allele underwent a selection to compensate such a hypermorphic allele involved in inflammatory-related gene in linkage disequilibrium. This has been proved in Keskitalo et al. where H232 compensate to a cis GOF mutation of STING^69^. Having provided supporting evidence for H232 as a hypomorphic *STING1* allele, a possible explanation for its selection comes from studies performed in STING KO mice infected with *Herpes* virus, where it was proposed that the challenge route is crucial for outcome. While infection through standard laboratory procedures (e.g., intravenous or intracerebral injection) il lethal for STING KO mice, the simulation of a natural entry site (e.g., mucosal infection) would compensate for STING loss via alternative inflammatory pathways protecting from brain injury^70,71^.

## Materials and methods

### Cell cultures manipulation and characterization

THP-1-Dual, THP-1-Dual KO-STING, THP-1-Dual KI-hSTING-H232, THP-1-Dual KI-hSTING-R232, THP-1-Dual KI-hSTING-M155, CT26, CT26_SKO and SKOV3 were cultured with RPMI 1640 Medium GlutaMAX™ Supplement (Gibco™, Thermo Fisher Scientific, Waltham, MA, USA). All the media were supplemented with 10% heat-inactivated fetal bovine serum and 50 UI/mL penicillin, 50 µg/mL streptomycin (Gibco™, Thermo Fisher Scientific, Waltham, MA, USA). Normocin [100 µg/mL] and 25 mM HEPES are required for THP-1 culture (Invivogen, San Diego, CA, USA). Catalog code: thpd-r232; Catalog code: thpd-h232; Catalog code: thpd-m155; Catalog code: thpd-nfis; Catalog code: thpd-kostg (Invivogen, San Diego, CA, USA). Initial culture of all THP-1 derived cell lines must be performed in growth medium containing 20% heat-inactivated FBS. After THP-1 cells have recovered, Blasticidin [10 µg/ml] and Zeocin™ [100 µg/ml] were added to the growth medium to maintain selection pressure of THP-1 cells (Invivogen, San Diego, CA, USA). CT26 cell line was purchased from ATCC cultured in a humidified atmosphere containing 5% CO2 at 37 °C. CT26 STING KO was described in Froechlich et al 2020^47^. CT26_hR232, CT26_hH232, CT26_hHAQ were generated starting from CT26_SKO cell line. CT26_SKO were plated in MW6 and transfected with expression vectors encoding hSTING alleles (pUNO1-hSTING-R232, pUNO1-hSTING-H232, pUNO1-hSTING-HAQ) by Lipofectamine 3000 (Thermo Scientific, Waltham, MA, USA). Two days after transfection, medium was supplemented with 5 µg/mL Blasticidin to select cells with stably incorporated pUNO1 plasmid of interest (Invivogen, San Diego, CA, USA). The validation of the reconstitution of *STING* isoforms was assessed by Western blot analysis. Filters were probed with the rabbit anti-STING antibody (Cell Signaling, Danvers, MA, USA, #13647), followed by anti-rabbit secondary antibody. Pierce™ ECL Western Blotting Substrate (Thermo Scientific, Waltham, MA, USA) was used for signal development, according to the manufacturer’s recommendations^72^.

### Nucleic acid transfection and activity assays

THP-1 cell lines were plated at 500.000 cell/mL in 6-well plate. At the same time, the cells were transfected as following: DNA [2 µg/ml], 5′ triphosphate hairpin RNA (3p-hp-RNA) [30 ng/ml] and 2′,3′cGAMP (cyclic [G (2′,5′pA (3′,5′) p]) [1 µg/ml] (Invivogen, San Diego, CA, USA). Transfection was performed using Lipofectamine reagent 3000 (Thermo Scientific, Waltham, MA, USA). After 24 h from the transfection, Luciferase and SEAP assays were performed according to QUANTI-Luc ™ and QUANTI-Blue Solution ™ protocols (Invivogen, San Diego, CA, USA). Immunofluorescence was performed as reported in Sasso et al.^72^.

### Virus production, titration and infection

HSV-1 BAC virus used in this article was described in Sasso et al.^73^. HSV-1_Δ34.5 was generated from wild type HSV-1 by BAC recombineering to recapitulate deletions in 34.5 and ICP47 as reported into the clinically approved T-VEC^74^. The viruses were produced and titrated in SKOV3 cells according as previously described^75^. To analyze viral replication, THP-1 cells were plated 500.000 cell/mL and incubated with HSV-1 strain F and HSV-1_Δ34.5 with at MOI 0.1 pfu/cell. After 96 h post infection, the bulk were collected and the viral replication was assessed by plaque-forming assay on SKOV3 cells as previously described^75^.

To perform IFN activity assay, THP-1 cell lines were plated 500.000 cell/mL in 6-well plate and infected with HSV-1 strain F at MOI 0.1 pfu/cell. The supernatants were collected at different time points and luciferase assays was performed according to QUANTI-Luc TM protocol (Invivogen, San Diego, CA, USA). The cytotoxicity of virus-infected cells was determined by measuring the release of extracellular LDH. THP-1 cells were plated 500,000 cell/mL in 6-well and infected with HSV-1 strain F at MOI 0.1 pfu/cell. The supernatants were collected at different time points and LDH was dosed by CyQUANT™ LDH Cytotoxicity Assay (Invitrogen, San Diego, CA, USA).

To evaluate viral spread, CT26_WT, CT26_SKO, CT26_R232, CT26_H232, CT26_HAQ cells were plated 800.000 cell/well in 12-well plate. The next day they were infected with BAC-HSV-1 derived from strain F and encoding eGFP at MOI 0.01 pfu/cell and 24 h and 48 h post infection. The spread of virus was assessed as eGFP + cells by fluorescence microscopy. To study cell viability, CT26_WT, CT26_SKO, CT26_R232, CT26_H232, CT26_HAQ cells were plated 800,000 cell/well in 12-well plate. The next day they were infected with BAC-HSV-1 at MOI 0.1 pfu/cell and 48 h post infection the percentage of live cell was obtained by counting live cells on the non-infected count.

### THP-1 cells differentiation and mRNA dosage

THP-1 cells were plated 500,000 cell/mL in 6-well plate, treated with PMA [50 ng/ml] (Invivogen, San Diego, CA, USA) and transfected with 2 µg/mL DNA using Lipofectamine 3000 (Thermo Scientific, Waltham, MA, USA). After 18 h, the cells were washed twice with PBS and were lysed by TriFast (Euroclone, Pero, Italy) and total RNA was extracted with phenol/chloroform. Then, 3 µg of RNA was treated with RQ1 RNase-free Dnase (Promega, Madison, WI, USA) and 1 µg of RNA was reverse-transcribed by using ImProm-II Reverse Transcriptase (Promega, Madison, WI, USA). The cDNA was then amplified using SYBR Green PCR Mastermix (Applied Biosystem, Foster City, CA, USA). The relative abundance of target RNAs was evaluated in relation to RPLP0 transcript by ΔΔCt method^76^.

### ELISA assay

To assess the production of IFNβ, CT26_WT, CT26_SKO, CT26_hR232, CT26_hH232, CT26_hHAQ cells were infected with HSV-1 at MOI 10 pfu/cell and MVA at MOI of 1 and 5. After 10 h and 48 h post infection, aliquots of supernatants were collected and IFNβ was dosed with Mouse IFNβ ELISA according to the manufacturer’s instructions (IFNβ, catalog no. 42400, PBL assay science Inc., NJ, USA).

### Clinical response to Nivolumab

The exome sequencing NGS data of pheripheral blood from 1 cohort of melanoma patients treated with anti-PD1, was downloaded from SRA database (bioproject IDs: PRJNA359359). The raw reads were preliminary subjected to quality control with FASTQC 0.12.0 and were trimmed with Trimmomatic 0.33^77^ with the following parameters (LEADING:5; TRAIL-INGri:5; SLIDINGWINDOW:4:20; MINLEN:50). Trimmed reads were then aligned to the hg38 human genome with BWA-MEM software^78^ with default parameters. Look up of rs1131769 in each patient was performed with an in-house Perl script that imple-ments the samtools-mpileup tool^79^ to estimate the number of mut/wt Exo-meseq reads at genomic locus of interest. Clinical response and days to death of the patients were retrieved from the original works. The Kaplan–Meier analysis was performed by using the survfit function included in survival 3.5.3 R pack-age (Therneau TM. A Package for Survival Analysis in R [Internet]. 2020. Available from: https://CRAN.R-project.org/package=survival).

### Supplementary Information

Supplementary Figures.

## Data Availability

All data, cell line models, constructs and viral vectors are available upon request to Emanuele Sasso emanuele.sasso@unina.it.

## Abbreviations

LOF: Loss of function
GOF: Gain of function
