# Supplementary material for: The common H232 STING allele shows impaired activities in DNA sensing, susceptibility to viral infection, and in monocyte cell function, while the HAQ variant possesses wild-type properties
Source: Sci Rep. 2023 Nov 9;13:19541. doi: 10.1038/s41598-023-46830-5 (PMC10636114; doi:10.1038/s41598-023-46830-5)
Supplement: Supplementary file 1 — Supplementary Figures. [file 41598_2023_46830_MOESM1_ESM.pdf]

Supplementary information

The common H232 STING allele shows impaired activities in DNA sensing, susceptibility to viral infection, and in monocyte cell function, while the HAQ variant possesses wild-type properties

Guendalina Froechlich[a,b], Arianna Finizio[a,b,x], Alessandra Napolano[a,b,x], Sara Amiranda[a,b], Arianna De Chiara[a,b], Pasqualina Pagano[a,b], Massimo Mallardo[a], Guido Leoni[c], *Nicola Zambrano[a,b], *Emanuele Sasso[a,b,d]

[a]Dipartimento di Medicina molecolare e Biotecnologie mediche, Università degli Studi di Napoli Federico II, Via Pansini 5, 80131 Napoli,NA, Italy; [b]CEINGE Biotecnologie Avanzate Franco Salvatore S.C.aR.L.., Via Gaetano Salvatore 486, 80145 Naples, Italy; [c]Nouscom S.R.L., Rome, Italy; [d]ImGen-T srl viale del parco carelli Napoli, NA, Italy.

Fig.S1 Characterization of THP-1 cell lines.

(A) Validation of HAQ, H232 and R232 alleles in THP-1 cell lines by Sanger Sequencing from retro transcribed RNA (cDNA). The images show the modified triplets highlighted in blue boxes. (B) Validation of STING absence in THP-1 SKO cell line by Western Blot analysis.

Fig.S2 IFNβ transcriptional activation in differentiated M155 macrophages.

THP-1 M155 cell lines were treated with phorbol myristate acetate (PMA) alone or with PMA in combination with a DNA stimulus. After 18h the transcriptional activation of endogenous IFNβ was evaluated into differentiated macrophages. The statistical analysis was performed by student's t test using NT as reference.

Fig.S3 Susceptibility of THP-1 cell lines to HSV-1 infection.

Cell viability in THP-1 R232, HAQ, H232, STING KO and M155 cell lines after HSV-1 and HSV-1_Δ34.5 infection at MOI 0.1 pfu/cell. The count of live cells was evaluated after 5 days post infection.

Fig.S4 Uncropped WB images

Fig.S5 Spread and IFNβ secretion in CT26 WT cell line.

(A) Spread of eGFP-encoding HSV-1 was evaluated by fluorescence microscopy in CT26 WT cell lines after 48h post infection. (B) CT26 WT cell line was infected at MOI 10 pfu/cell with wild type HSV-1 and the supernatants were collected two days post infection to dose interferon beta (left panel). The statistical analysis was performed by student's t test using NI as reference. The same experiment was performed using a different DNA-based virus, MVA at MOI 1 and 5. The statistical analysis was performed by student's t test using NI as reference.

Fig.S6 IRF3 localization.

Fig. S7 Monocyte differentiation.

The images show monocyte differentiation in THP-1 R232 and SKO cell line after stimulation with DNA.

Fig. S8 CD86-CD163-NLRC5 transcriptional activation in differentiated M155 macrophages.

THP-1 M155 cell lines were treated with phorbol myristate acetate (PMA) alone or with PMA in combination with a DNA stimulus. After 18h the transcriptional activation of endogenous CD86, CD163 and NLRC5 were evaluated into differentiated macrophages. The statistical analysis was performed by student's t test using NT as reference.

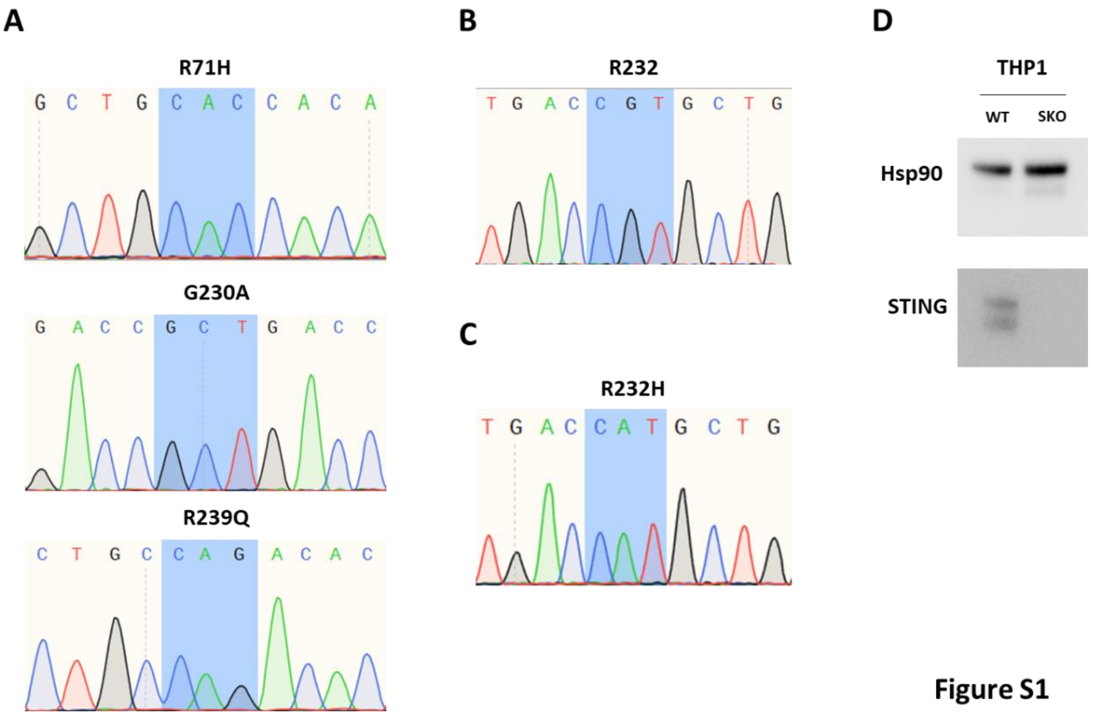

Figure S1

IFNβ transcript

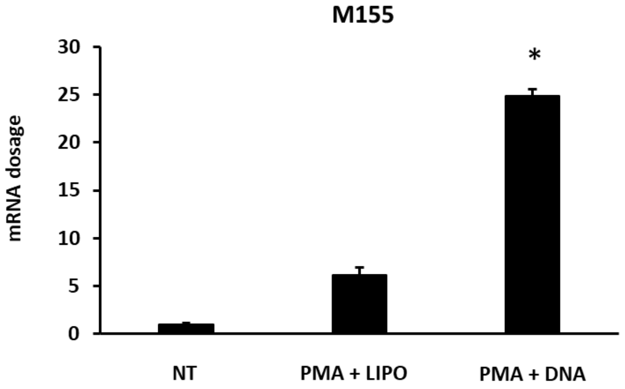

Figure S2

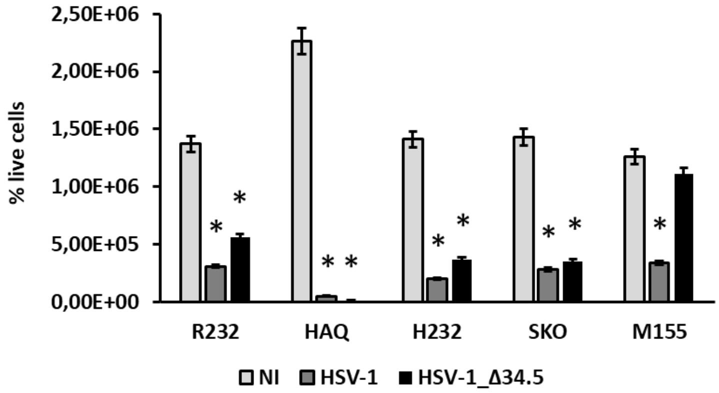

Figure S3

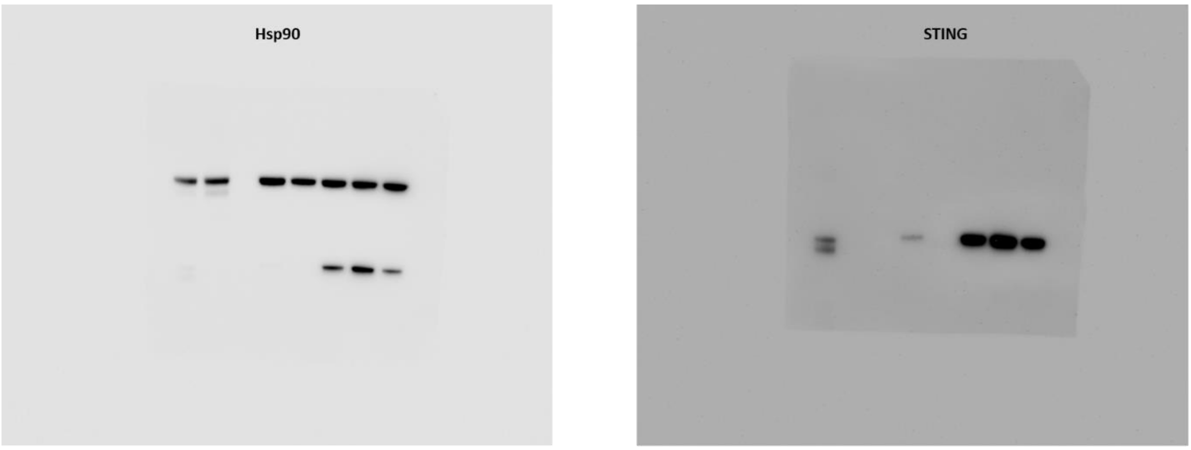

Figure S4

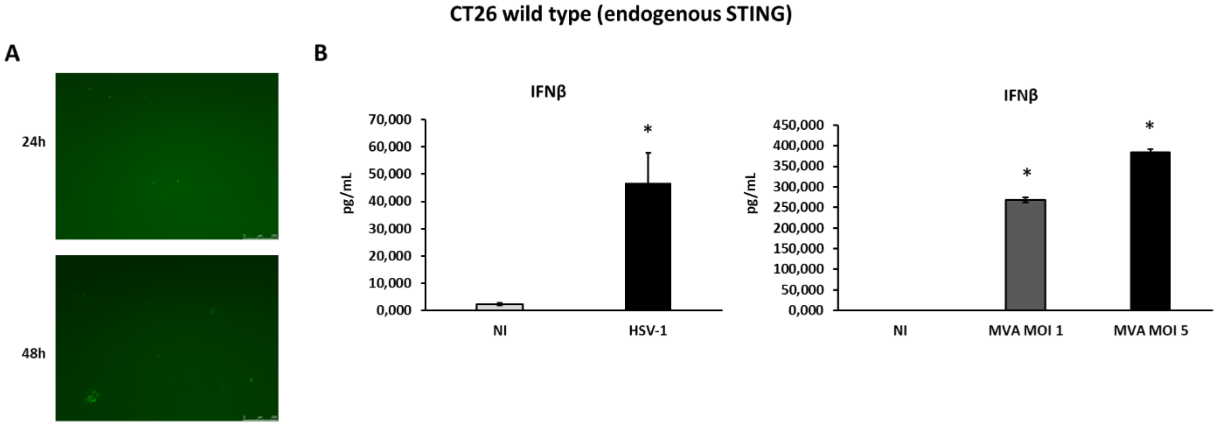

Figure S5

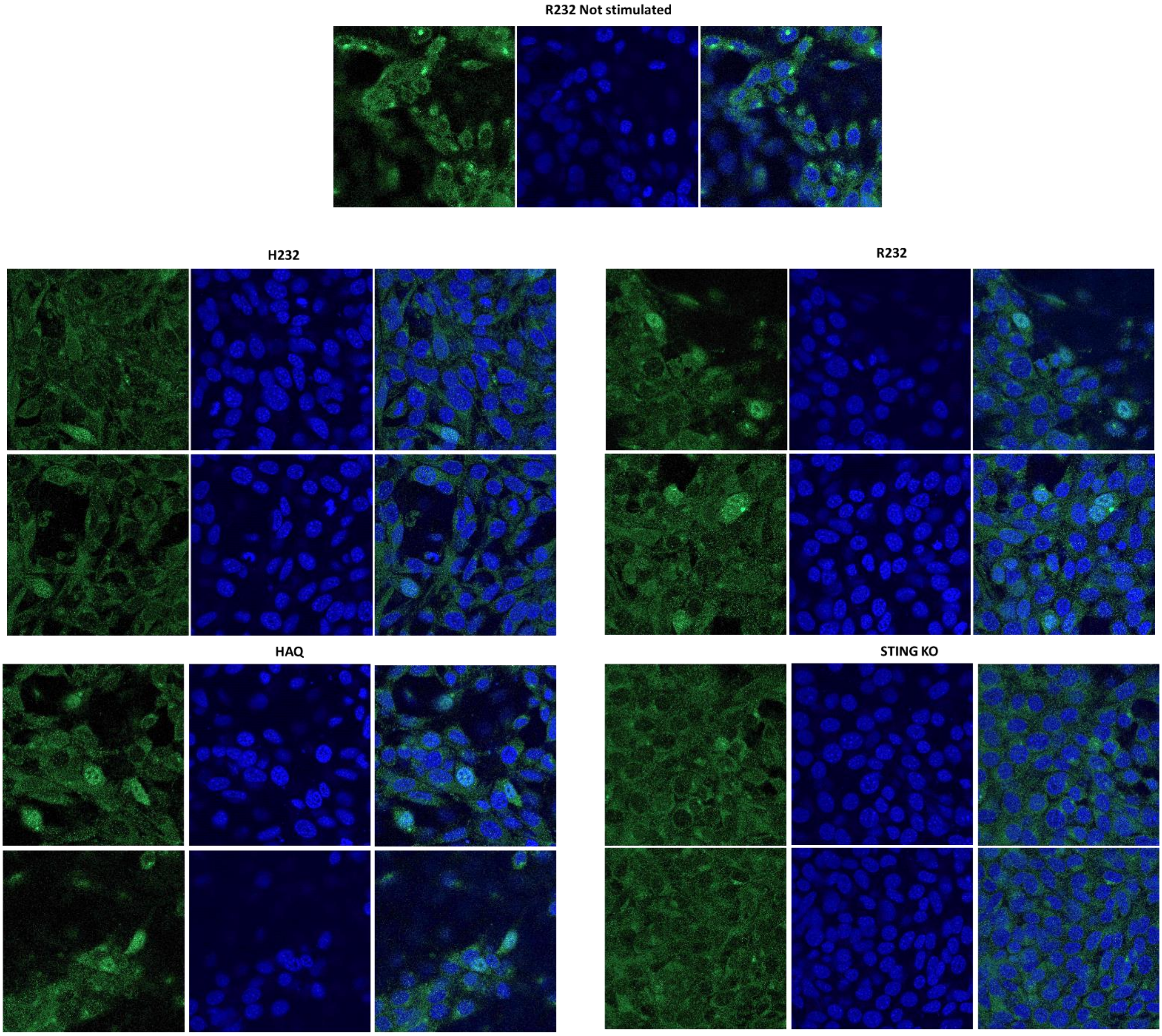

Figure S6

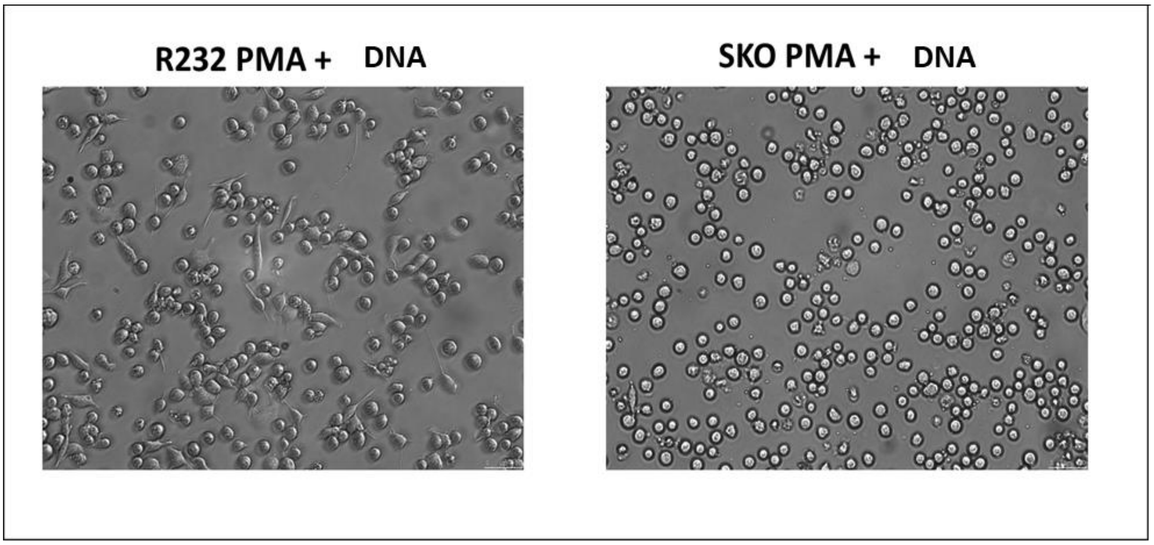

Figure S7

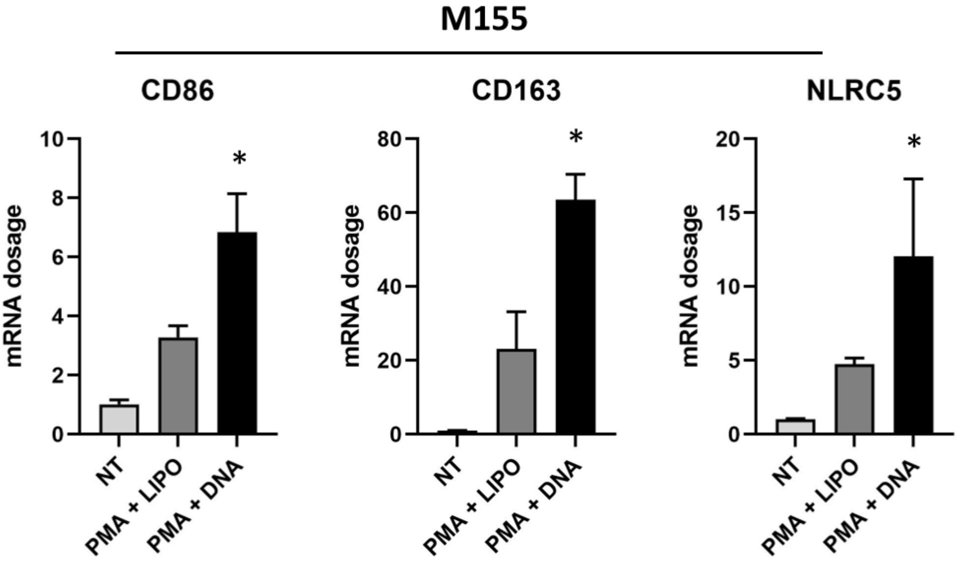

Figure S8
